# A comparative analysis between NAT and chemiluminescence in detection of transfusion transmitted viruses in two main university blood transfusion centers

**DOI:** 10.1038/s41598-025-03506-6

**Published:** 2025-06-20

**Authors:** Doaa Shahin, Rabab Aly, Mayada Ghannam, Omnia Khaled, Mona Sadeq, Ahmed Elzeiny, Youssef Mosaad, Mariam Abdallah, Nada abdelhameed, Eman NasrEldin

**Affiliations:** 1 Clinical Hematology Unit, Clinical Pathology Department, Faculty of Medicine, Mansoura University, Mansoura, 35111 Egypt; 2 Clinical Immunology Unit, Clinical Pathology Department, Faculty of Medicine, Mansoura University, Mansoura, Egypt; 3 Clinical Hematology Unit, Clinical Pathology Department, Faculty of Medicine, Assiut University, Assiut, Egypt

**Keywords:** Chemiluminescence, NAT, HCV, HBV, HIV, Screening, Transfusion, Egyptian, Immunological techniques, Laboratory techniques and procedures

## Abstract

Nucleic acid amplification Testing (NAT) is expected to minimize the potential risk of transfusion transmitted infections (TTIs) which escaped the detection by serologic screening. Therefore, the aim was to assess the seroprevalence of TTIs (HCV, HBV and HIV) in voluntary blood donors and to verify the accuracy of chemiluminescence (CLIA) test versus NAT as confirmatory test for more safe blood donations in two main university (Mansoura and Assiut) blood transfusion centers in Egypt. A retrospective analysis was done for 87,620 specimens from healthy voluntary blood donors by NAT and CLIA techniques in Mansoura (12,464) and Assiut (75,156) blood transfusion centers. The prevalence of viral reactivity by CLIA was 2.49% (0.77%, 1.69% and 0.03% for HBV, HCV and HIV respectively) and by NAT was 2.25% (HBV 0.71%, HCV 1.52% and HIV 0.02%). The CLIA seroreactivity for HBV was found in 676 samples versus 621 by NAT (589 true positive, 87 false positive and 32 false negative). HCV reactivity was detected in 1477 by CLIA versus 1328 by NAT (1305 true positive, 172 false positive and 23 false negative). HIV results showed (CLIA/NAT) 22 versus 19 reactive samples (19 true positive, 3 false positive and 0.0 false negative). Comparison of CLIA and NAT results as regards the accuracy revealed 99.86% ,99.78%, and 100% for HBV, HCV, and HIV respectively. The CLIA and NAT techniques showed perfect agreement for detection of HCV (kappa = 0.929), HBV detection (kappa = 0.907) and HIV detection (kappa = 0.900). NAT implementation with CLIA increased blood transfusion safety with the advantage of direct sequence specific detection of virus genome. Therefore, it is a critical role to adopt NAT technology in all blood transfusion centers in Egypt.

## Introduction

Although blood transfusion has saved millions of lives all over the world, transfusion transmitted infections (TTIs) still represent a major hazard to blood recipients. The four major TTIs are Hepatitis B virus (HBV), Hepatitis C virus (HCV), Human immunodeficiency virus (HIV), and Treponema pallidum (TP)^1,3^.

According to the World Health Organization (WHO) 2021^4^ report, about 118.5 million blood donations were made (either whole blood or apheresis donations). The blood units were collected from voluntary, family, and paid donations. There are about 296, 58.0, and 39.0 million persons infected by HBV, HCV, and HIV respectively and the prevalence of such viruses in blood donors ranged from (high-income-low-income countries) 0.02 to 6.02%, 0.007 to 1.67%, and 0.002 to 1.6%, respectively. WHO planned to eliminate TTIs by screening blood donations in a quality-based manner and targeted to be zero risk in 2030^4,5^.

The starting point for safe blood supply is the regular voluntary donations which represent 82.8% globally (about 95.6% in high-income countries versus 62.8% in low-income countries). The least risk of TTIs is associated with repeat voluntary donations and the risk can be reduced by systematic screening and testing for TTI markers. The use of standard techniques and active participation in external quality assessment are very crucial in this issue^4–6^.

The CLIA uses a chemical or enzymatic reaction to measure the absolute concentration of pathogen antibody or antigen in blood sample (i.e. measures the immune response of donors to viruses) by measuring the luminescence intensity. The NAT detects directly the nucleic acid of viruses by molecular biology technique (i.e. amplify targeted regions of viral nucleic acid before appearance of viral antigen or antibody). Therefore, the sensitivity and specificity of NAT are higher than those of CLIA, and the window between infection and detection of viruses by NAT is shorter than CLIA^7,8^ The CLIA advantages include short time assay, low sample cost, high throughput, and simple equipment and technique. The NAT advantages include minimizing the risk of contamination, identifying different even emerging new viruses, and with a significant impact on blood safety (i.e. reduces the risk of TTIs from these viruses). However, NAT has some limitations such as pool assay which may need to reassess samples when the pool is positive, high cost, long time testing, needs specialized infrastructure, specialized consumables and equipment^9,14^.

Cadena-Ullauri et al.^9^ documented the improvement in blood safety due to implementation of NAT in routine screening for TTIs. They recommended the global use of NAT in blood transfusion protocols, especially the countries with high prevalence of viral infection that have not yet applied this technology to provide safe blood for everyone. Gadji et al.^8^ study on population from sub–Saharan Africa concluded that CLIA may be associated with false positive results in addition to the problem with viral window phase and recommended the use of NAT as a screening or confirmatory test. Therefore, this study aimed to assess the seroprevalence of TTIs in voluntary blood donors and to verify the accuracy of CLIA test versus NAT as confirmatory test for more safe blood donations in two main university blood transfusion centers in Egypt.

## Material and method

A retrospective analysis was done through the period from 2019 to 2023 at two main blood centers in Egypt: Assiut University from July 2019 to March 2023 and Mansoura University from October 2021 to March 2023. The study was conducted on 87,620 blood specimens from healthy voluntary blood donors (75,156 donors from Assiut and 12,646 donors from Mansoura). The gender of blood donors was 80,847 males (92.3%) and 6771 females (7.7%). The type of blood donation was regular (voluntary non-remunerated blood donor who has donated at least three times, the last donation being within the previous year, and continues to donate regularly at least once per year), and non-regular (family/replacement donor who donates blood in replacement of blood needed for a member of his/her family or community). They were a mixture of first-time donors or known donors repeating donations more than a year after previous donation (relapsed donors).

The distribution of male/female ratio of blood donors was 92.5/7.5% for Assiut and 90.9/9.1% for Mansoura. The age range was between 18 and 55 years. A detailed pre-donation questionnaire was taken and medical history for recent donation within 3 months, recent surgeries, history of blood transfusion and history of chronic illness and clinical examination including measuring of both blood pressure and hemoglobin concentration. This study protocol was reviewed and approved by Mansoura faculty of medicine local ethical committee, approval number [R.23.12.2410]. Informed consent was obtained from each donor.

### Serological assay

All serological investigation was done according to the manufacturer’s instruction. HBs antigen, HCV antibody, HIV antigen and antibody were tested by CLIA kits using the Architect i2000SR immunoassay analyzer (Abbott, Wiesbaden, Germany). The Architect Anti-HCV CIMA assay kit was used for the qualitative detection of HCV-IgG and IgM antibodies. The system calculated results by S/Co ratio (Sample RLU/Cutoff RLU ratio). The CO is calculated by multiplying the mean of 3 measured calibrator one by 0.074. Samples with S/CO value less than 1.0 are considered nonreactive.

The Architect HBsAg qualitative II assay kit was used for qualitative detection of HBs antigen. The system calculated results by S/Co ratio. The CO is calculated by multiplying the mean of 3 measured calibrator one by 0.0575 and calibrator 2 by 0.8. Samples with S/CO value less than 1.0 are considered nonreactive. The HIV screening was done by Architect HIV Ag/Ab Combo assay kit (simultaneous detection of HIV antigen and antibodies). The results were calculated by S/Co ratio (CO = mean of calibrator oneX0.04). Samples with S/CO value less than 1.0 are considered nonreactive.

### NAT testing

All blood units were subjected to NAT for HBV-DNA, HCV-RNA, and HIV-RNA. NAT was done in Mansoura University by multiplex HIV, HCV & HBV nucleic acid test using Cobas 6800/8800 MPX 480 T CE-IVD (Roche Diagnostics, Rotkreuz, Switzerland) and was done in Assiut University by Procleix Ultrio assay (Gen-Probe, CA, USA). The NAT testing was done on every individual sample by individual donor nucleic acid testing (ID – NAT). The test procedure was done according to the manufacturer’s instructions.

The cobas^®^ MPX test is a qualitative multiplex test that enables the simultaneous detection and discrimination of HIV RNA, HCV RNA, HBV DNA, and the internal control in a single test. It is fully automated real time PCR technology (automated nucleic acid extraction, purification, amplification and detection). The system consists of many modules, sample supply, transfer, processing, and analytical. Automated data management software assigns the test results for all tests as non-reactive, reactive, or invalid.

HCV interpretation of NAT results with CLIA results was done as follows; sera were considered 1- True Positive: anti-HCV antibodies positive by CLIA and positive by NAT (current active infection), 2- False Positive: anti-HCV antibodies positive by CLIA with negative NAT (consistent with spontaneous clearance or prior HCV treatment which is common in countries with high prevalence of HCV as Egypt), 3- True Negative : anti-HCV antibodies are indeterminate by CLIA and negative NAT (No HCV infection), and 4- False Negative: anti-HCV antibodies negative by CLIA and positive by NAT (acute infection acquired very recently).

HBV interpretation of NAT results with CLIA results was done as follows; sera were considered 1- True Positive: HBsAg positive by CLIA and positive by NAT (HBV infection), 2- False Positive: HBsAg antibodies positive by CLIA with negative NAT (transient positive HBsAg after vaccination), 3- True Negative : HBsAg are indeterminate by CLIA and negative NAT (not infected or immune from vaccination), and 4- False Negative: HBsAg negative by CLIA and positive by NAT (window period or occult HBV infection).

HIV interpretation of NAT results with CLIA (ARCHITECT HIV Ag/Ab Combo)results was done as follows; sera were considered 1- True Positive: HIV Ag/Ab Combo positive by CLIA and positive by NAT (current active infection), 2- False Positive: HIV Ag/Ab Combo positive by CLIA with negative NAT (due to presence and cross-reactivity of other antibodies as schistosomiasis, Epstein-Barr virus, SARS-CoV-2 in the serum of donors), 3- True Negative : HIV Ag/Ab Combo are indeterminate by CLIA and negative NAT (No HIV infection), and 4- False Negative: HIV Ag/Ab Combo negative by CLIA and positive by NAT (acute recent HIV infection).

### Statistical analysis

The Statistical Package for Social Sciences (SPSS Inc., Chicago, Illinois, USA, version 23.0) was used for statistical analysis. Qualitative data will be expressed as N and percentage (%). The diagnostic sensitivity: true positive/true positive + false negative (TP/TP + FN), specificity: true negative/true negative + false positive (TN/TN + FP), positive predictive value (PPV = TP/TP + FP), negative predictive value (NNP = TN/TN + FN) and accuracy of an index test (e.g., CLIA) were compared to the gold standard test (NAT). Confidence intervals for sensitivity, specificity and accuracy are “exact” Clopper-Pearson confidence intervals. Confidence intervals for the predictive values are the standard logit confidence interval given by Mercaldo et al.^15^ except when the predictive value is 0 or 100%, in which case a Clopper-Pearson confidence interval is reported^16^ Cohen’s Kappa test was used to assess the agreement between two modalities (e.g., CLIA and NAT) for measuring a categorical variable. Different classifications have been suggested for assessing how good the strength of agreement is when based on the value of Cohen’s kappa (κ) coefficient (< 0.20 poor, 0.21–0.40 fair, 0.41–0.60 moderate, 0.61–0.80 good and 0.81–1.00 perfect).

## Results

The demographic details of donors are given in Table 1. About 92.3% (80849) of blood donors were male. Most donors were young in the third decade of life, 44.7% (39168), and 33.5% (29352) in the fourth decade of life. The percent of regular blood donation was 5.9%, non-regular donation was 94.1%, and first-time blood donation was 26.7%. The most common donated blood group was A (34.7%), then O (30.4%), and B (25.2%). The AB was the least donated blood group (9.7%). As regards the Rh type, 91.5% of blood donors were Rh positive.

**Table 1:** Demographic data of donors among two main blood centers.

		Total (*N* = 87,620)	Assuit (*N* = 75,156)	Mansoura (*N* = 12,464)
Age group	< 20 (N/%)	1226/1.4	977/1.3	249/2.0
	20–30 (N/%)	39,168/44.7	33,820/45.0	5348/42.9
	30–40 (N/%)	29,352/33.5	24,350/32.4	5002/40.1
	40–50 (N/%)	14,282/16.3	12,876/17.1	1406/11.3
	> 50 (N/%)	3592/4.1	3133/4.2	459/3.7
Gender	Male (N/%)	80,849/92.3	69,519/92.5	11,330/90.9
	Female (N/%)	6771/7.7	5637/7.5	1134/9.1
Previous donation	Regular donor (N/%)	5169/5.9	3757/5.0	1412/11.3
	Non regular (N/%)	82,451/94.1	71,399/95.0	11,052/88.7
1 st time donation		23,394/26.7	19,037/25.3	4357/34.9
Blood grouping	A+ (N/%)	28,131/32.1	23,449/31.2	4682/37.6
	O+ (N/%)	24,453/27.9	20,668/27.5	3785/30.4
	B+ (N/%)	20,420/23.3	18,263/24.3	2157/17.3
	AB+ (N/%)	7153/8.2	6313/8.4	840/6.7
	A- (N/%)	2282/2.6	1954/2.6	328/2.6
	O- (N/%)	2193/2.5	1954/2.6	239/1.9
	B- (N/%)	1669/1.9	1428/1.9	241/1.9
	AB- (N/%)	1319/1.5	1127/1.5	192/1.6

**Fig. 1:**
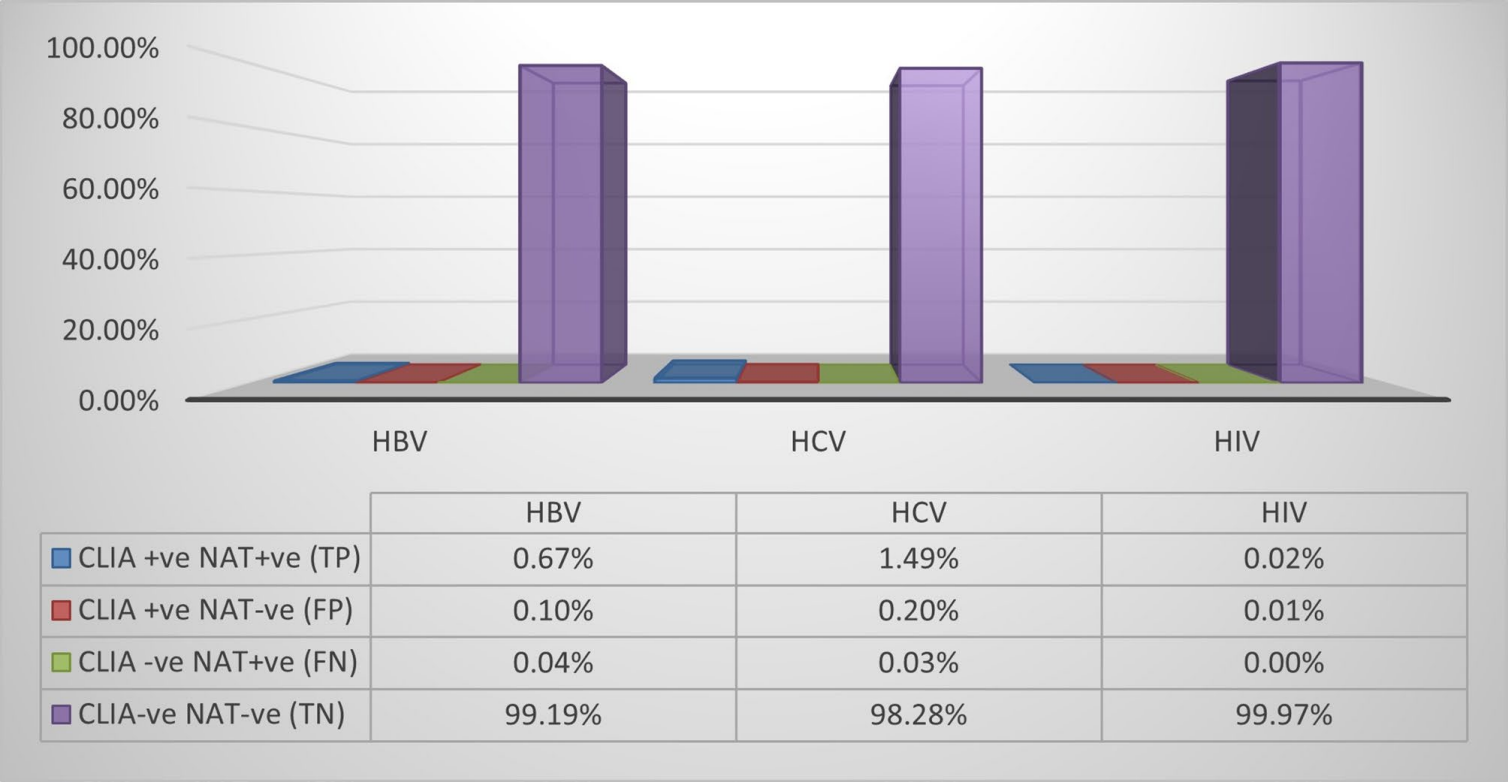
The figure illustrated the true positive (TP), false positive (FP), True negative (TN) and false negative (FN) results regarding HBV, HCV and HIV as regard analysis of specimens from healthy blood donors (*N* = 87620) by both chemiluminescent immunoassay (CLIA) and nucleic acid test (NAT).

**Fig. 2:**
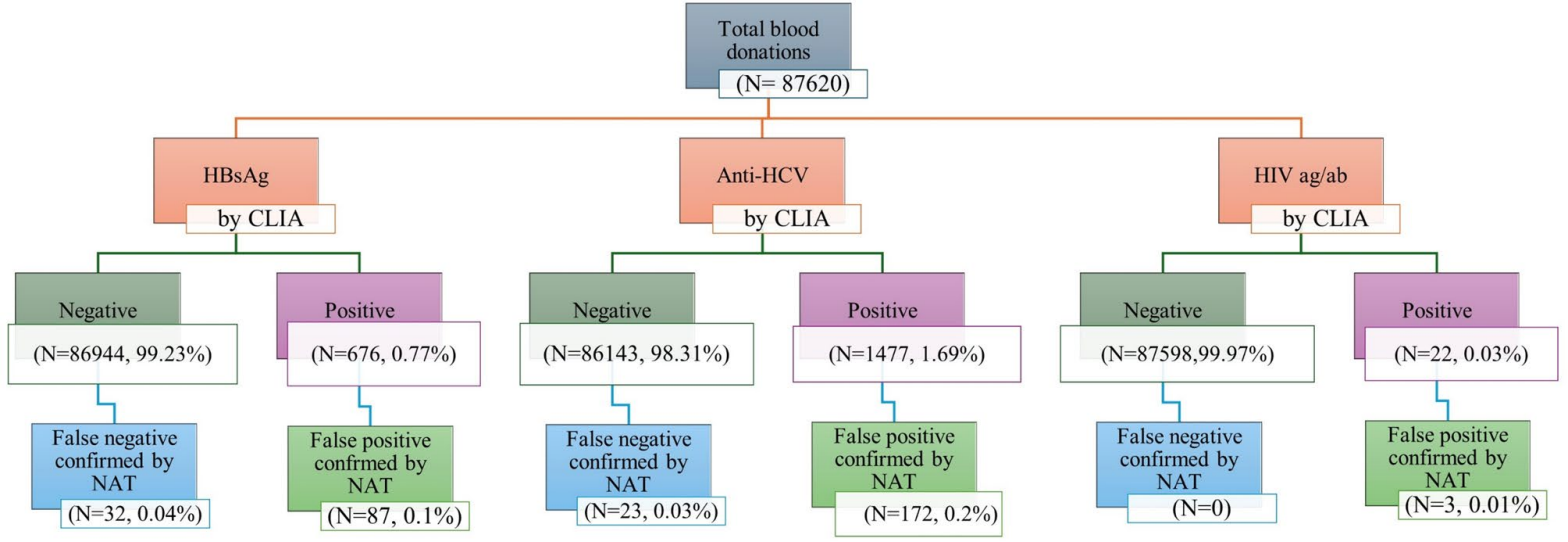
The figure illustrated the prevalence of reactivity of HBV, HCV and HIV by CLIA (0.77%, 1.69% and 0.03% respectively). All specimens’ results confirmed by NAT as gold standard method, which revealed false negative results **NAT yield** (0.04% for HBV, 0.03% for HCV) and false positive results (0.1% for HBV, 0.2% for HCV and 0.01% for HIV).

CLIA results showed non-reactivity in 97.51% of blood units (85,445). The 2.49% seropositive units by CLIA were classified as follows, 0.77% (676 units) positive for HBs antigen, 1.69% (1477 units) positive for HCV antibody, and 0.03% (22 units) positive for HIV antibody. As regards NAT results, the non-reactivity was found in 97.75% of blood units (85,652). This means that about 207 units were false positive results by CLIA. The distribution of false positive results by CLIA which were corrected by NAT was HBs antigen 55 units, HCV antibody 149 units, and HIV antibody 3 units. When stratifying the results according to the University, the reactivity in Assiut was reported in 0.8% versus 0.74% (CLIA/NAT) for HBs antigen, 1.73 versus 1.55% for HCV, and 0.02 versus 0.018% for HIV. As regards the results of Mansoura University, 0.6% versus 0.54% (CLIA/NAT) for HBs antigen, 1.44% versus 1.28% for HCV, and 0.05 versus 0.03% for HIV. (Table 2; Figs. 1 and 2)

**Table 2:** Prevalence of reactivity of HBV, HCV, HIV by chemiluminescence (CLIA) and nucleic acid testing (NAT).

		Total (*N* = 87620)		Assuit (*N* = 75156)		Mansoura (*N* = 12464)	
		CLIA	NAT	CLIA	NAT	CLIA	NAT
HBV	Reactive	676/0.77	621/0.71	601/0.8	553/0.74	75/0.6	68/0.54
HCV	Reactive	1477/1.69	1328/1.52	1298/1.73	1168/1.55	179/1.44	160/1.28
HIV	Reactive	22/0.03	19/0.02	16/0.02	14/0.018	6/0.05	5/0.04
Non-reactive		85,445/97.51	85,652/97.75	73,241/97.45	73,421/97.692	12,204/97.91	12,231/98.14

### Comparison of HBV by CLIA and NAT

Analysis of HBs antigen reactive units in both Universities revealed that the true positive (TP) units (positive CLIA and NAT) were 589/0.67%, false positive (FP) units (positive CLIA and negative NAT) were 87/0.1% units, false negative (FN) units (negative CLIA and positive NAT) were 32/0.04%, and the true negative (TN = negative CLIA and NAT) were 86,912/99.19%. These results showed 94.85% sensitivity, 99.9% specificity, 99.86% accuracy, and a very good 0.907 Kappa coefficient value. The distribution in Assiut was 525/0.7% TP, 76/0.1% FP, 28/0.04% FN, and 74,527/99.16% TN with 94.94% sensitivity, 99.9% specificity, 99.86% accuracy, and a very good 0.909 Kappa coefficient value. In Mansoura University, TP was 64/0.51%, FP was 11/0.09%, FN was 4/0.3% and TN was 12,385/99.37% with 94.12% sensitivity, 99.91% specificity, 99.88% accuracy, and very good 0.895 Kappa coefficient value (Table 3; Figs. 1 and 2).

**Table 3:** Prevalence of reactivity of HBV by chemiluminescence (CLIA) and nucleic acid testing (NAT).

HBsAg (*N*/%)	Total (*N* = 87620)		Assuit (*N* = 75156)		Mansoura (*N* = 12464)	
	CLIA	NAT	CLIA	NAT	CLIA	NAT
Non-reactive	86,944/99.23	86,999/99.29	74,555/99.2	74,603/99.26	12,389/99.4	12,396/99.46
Reactive	676/0.77	621/0.71	601/0.8	553/0.74	75/0.6	68/0.54
HBsAg + ve NAT + ve (TP)	589/0.67		525/0.7		64/0.51	
HBsAg + ve NAT-ve (FP)	87/0.1		76/0.1		11/0.09	
HBsAg -ve NAT + ve (FN) **(NAT yield)**	32/0.04		28/0.04		4/0.03	
HBsAg -ve NAT-ve (TN)	86,912/99.19		74,527/99.16		12,385/99.37	
Sensitivity, Specificity, PPV, NPV and accuracy of chemiluminescence compared to gold standard test NAT (value, 95% CI)						
Sensitivity	94.85% (92.80–96.45%)		94.94% (92.77–96.61%)		94.12% (85.62–98.37%)	
Specificity	99.90% (99.88–99.92%)		99.90% (99.87–99.92%)		99.91% (99.84–99.96%)	
PPV	87.13% (84.58–89.32%)		87.35% (84.65–89.64%)		85.33% (76.27–91.33%)	
NPV	99.96% (99.95–99.97%)		99.96% (99.95–99.97%)		99.97% (99.92–99.99%)	
Accuracy	99.86% (99.84–99.89%)		99.86% (99.83–99.89%)		99.88% (99.80–99.93%)	
Inter-rater agreement - % - Kappa coefficient value	99.86% 0.907 (very good)		99.86% 0.909 (very good)		99.88% 0.895 (very good)	

### Comparison of HCV by CLIA and NAT

Analysis of HCV reactivity (both Universities) revealed 1305 TP units, 172 FP units, 23 FN units, and 86120 TN units with 98.27% sensitivity, 99.8% specificity, 99.78% accuracy, and a very good 0.929 Kappa coefficient value. The HCV results distribution in Assiut was 1149 TP units, 149 FP units, 19 FN units, and 73839 TN (98.37% sensitivity, 99.8% specificity, 88.52% 99.78% accuracy, and 0.930 Kappa coefficient value. The Mansoura University results revealed 156 units were TP, 23 units were FP, 4 units were FN, and 12281units were TN (97.5% sensitivity, 99.81% specificity, 99.78% accuracy, and 0.919 Kappa coefficient value. (Table 4; Figs. 1 and 2)

**Table 4:** Prevalence of reactivity of HCV by chemiluminescence (CLIA) and nucleic acid testing (NAT).

HCV (*N*/%)	Total (*N* = 87620)		Assuit (*N* = 75156)		Mansoura (*N* = 12464)	
	CLIA	NAT	CLIA	NAT	CLIA	NAT
Non-reactive	86,143/98.31	86,292/98.48	73,858/98.27	73,988/98.45	12,285/98.56	12,304/98.72
Reactive	1477/1.69	1328/1.52	1298/1.73	1168/1.55	179/1.44	160/1.28
Anti-HCV + ve NAT + ve (TP)	1305/1.49		1149/1.53		156/1.25	
Anti-HCV + ve NAT-ve (FP)	172/0.2		149/0.2		23/0.19	
Anti-HCV -ve NAT + ve (FN) **(NAT yield)**	23/0.03		19/0.03		4/0.03	
Anti-HCV -ve NAT-ve (TN)	86,120/98.28		73,839/98.24		12,281/98.53	
Sensitivity, Specificity, PPV, NPV and accuracy of chemiluminescence compared to gold standard test NAT (value, 95% CI)						
Sensitivity	98.27% (97.41–98.90%)		98.37% (97.47–99.02%)		97.50% (93.72–99.31%)	
Specificity	99.80% (99.77–99.83%)		99.80% (99.76–99.83%)		99.81% (99.72–99.88%)	
PPV	88.35% (86.73–89.81%)		88.52% (86.79–90.05%)		87.15% (81.84–91.08%)	
NPV	99.97% (99.96–99.98%)		99.97% (99.96–99.98%)		99.97% (99.91–99.99%)	
Accuracy	99.78% (99.74–99.81%)		99.78% (99.74–99.81%)		99.78% (99.68–99.86%)	
Inter-rater agreement - % - Kappa coefficient value	99.77% 0.929 (very good)		99.77% 0.930 (very good)		99.78% 0.919 (very good)	

### Comparison of HIV by CLIA and NAT

While the reactivity of HIV testing by CLIA in both Universities, Assiut and Mansoura were 22, 16 and 6 samples respectively, the NAT results were 19, 14 and 5 respectively. The sensitivity was 100% for all, the specificity was100%,100% and 99.99% respectively, the accuracy was 100%,100% and 99.99% respectively, and the Kappa coefficient value were 0.900, 0.933 and 0.909 respectively (Table 5; Figs. 1 and 2).

**Table 5:** Prevalence of reactivity of HIV by chemiluminescence (CLIA) and nucleic acid testing (NAT).

HIV (*N*/%)	Total (*N* = 87620)		Assuit (*N* = 75156)		Mansoura (*N* = 12464)	
	CLIA	NAT	CLIA	NAT	CLIA	NAT
Non-reactive	87,598/99.97	87,601/99.98	75,140/99.98	75,142/98.982	12,458/99.95	12,459/99.96
Reactive	22/0.03	19/0.02	16/0.02	14/0.018	6/0.05	5/0.04
HIVAg/Ab + ve NAT + ve (TP)	19/0.02		14/0.018		5/0.04	
HIVAg/Ab + ve NAT-ve (FP)	3/0.01		2/0.002		1/0.01	
HIVAg/Ab -ve NAT + ve (FN) **(NAT yield)**	0/0.0		0/0.0		0/0.0	
HIVAg/Ab -ve NAT-ve (TN)	87,598/99.97		75,140/99.98		12,458/99.95	
Sensitivity, Specificity, PPV, NPV and accuracy of chemiluminescence compared to gold standard test NAT (value, 95% CI)						
Sensitivity	100.0% (81.47–100.0%)		100.0% (76.84–100.0%)		100.0% (47.82–100.0%)	
Specificity	100.0% (99.99–100.0%)		100.0% (99.99–100.0%)		99.99% (99.96–100.0%)	
PPV	81.82% (62.81–92.30%)		87.50% (63.65–96.55%)		83.33% (41.33–97.26%)	
NPV	100.0% (99.9–100.0%)		100.0% (99.9–100.0%)		100.0% (99.97–100.0%)	
Accuracy	100.00% (99.99–100.0%)		100.00% (99.99–100.0%)		99.99% (99.96–100.0%)	
Inter-rater agreement - % - Kappa coefficient value	99.99% 0.900 (very good)		99.99% 0.933 (very good)		99.99% 0.909 (very good)	

## Discussion

Although blood transfusion nowadays is more safe than before, still it remains a major public health concern. The use of more sensitive techniques for screening the blood units has reduced the risk of TTIs worldwide^10^ However, some infected blood units, due to very low viral load, viral variation, sero-conversion or operator error, may be missed during routine screening test^11^ The WHO planned a 2030 strategy to reach zero risk of TTIs. This issue raised the need for more sensitive and specific techniques to enable the detection of several viral specific antigens, antibodies, and genomes^12^ Implementation of both NAT and CLIA for this issue is efficient to achieve this target^13^. While, CLIA is characterized by higher stability and accuracy than ELISA, the NAT screening is the best for viral detection during window phase before seroconversion^10^.

The present study was carried out on 87,620 donor blood samples. To the best of our knowledge, this study may be the first on Egyptian to be done on this large number of blood samples when compared with other Egyptian studies such as Ebeid et al.^14^ (1000 samples), Attia et al.^17^ (2132 samples), and Kamel and Rageh^18^ (53138 samples). The prevalence of viral reactivity in the present work is 2.49% by CLIA and the viral distribution is 0.77 for HBV, 1.69% for HCV and 0.03% for HIV. This prevalence is comparable with reported prevalence in Egyptian by Sayed et al.^19^ (HIV 0.23%, HBV 0.76%, and HCV 2.33%), and Kamel and Rageh^18^ (HCV 1.87% and HBV 0.97%). In contrast, the reported prevalence per 100 000 donations in the USA was 27.9% (HIV 2.65%, HBV 6.3%, and HCV 19.0%)^20^.

In economically restricted countries, still the supply of safe blood is major health problem (i.e. inadequate infrastructure, lack of economic and political resources)^8^ In Africa, the prevalence of TTIs was 5.0–10.0% for HIV and about 12.5% for viral hepatitis^21^ The reported prevalence of TTIs in Ghana^21^ (21%), Eritrea^22^ (3.6%), Nigeria^23^ (9.83%), Tanzania^24^ (10.1%), Kenya^25^ (9.4%), Malawi^26^ (10.7%) and Ethiopia^27^ (11.5%). However, the pooled estimates for TTIs were 2.0% in the systematic review of Puerto-Meredith et al.^28^ on populations (i.e. donors from 16 nations) from Southern African Development Community (SADC). They also reported the pooled prevalence of 2.0%, 3.0% and 1.0% for HIV, HBV, and HCV respectively. Also, the prevalence reported by Gadji et al.^8^ (donors from southern Saharan region) was 8.39%, 0.56%, and 0.1% for HBV, HIV, and HCV respectively. At the same time, the reported prevalence in Mozambique was 4.6%, 4.5%, and 0.4% for HIV, HBV, and HCV respectively^11^ The difference in the reported prevalence in the present work can be explained by the voluntary health donors are the main source of blood donation (low incidence of TTIs), obligatory vaccination strategies for HBV, increased awareness about the risk of drug abuse and needle sharing for viral transmission, number of samples in different studies, and different methods of viral testing.

The seroreactivity for HBs antigen in the total units (87,620) was found in 676 samples (0.77%) by CLIA (601 samples from Assiut and 75 samples from Mansoura) and the number was decreased by NAT to 621/0.71% (553 from Assiut and 68 from Mansoura). Comparing results of CLIA versus NAT revealed the presence of TP result in 589 units (positive CLIA and NAT), FP results in 87 units (positive CLIA and negative NAT), and FN results in 32 units (negative CLIA and positive NAT). This means that the use of NAT saved 87 positive CLIA units from being discarded (FP) and picked up sero-silent blood donation, thereby correcting the result of the 32-miss passed CLIA units from transfusion to patients and transmission of HBV (FN). However, comparison of CLIA results versus NAT results revealed 94.85% sensitivity, 99.9% specificity, and very good (0.907) Kappa coefficient value. Jin-feng et al.^27^ documented that the consistency of NAT and serology was very good for HBV (kappa = 0.815), and the results of CLIA are better than ELISA when compared with NAT.

The reactivity of HBsAg was 14 samples (1.4%) in Ebeid et al.^14^ (HBs antigen was tested by ELISA not CLIA) and was decreased to 6 samples only when was tested by NAT (0.6%). The Wasfi^30^ study on 3420 blood units reported the number of positive units to be 47 (1.4%) and the test was done by ELISA. The low prevalence in the present study can be explained by sample size, screening technique and progressive program of HBV vaccination and pre-donation screening which excludes those known to be at high risk of contracting bloodborne infections.

Several studies have documented the effectiveness of HBV screening by NAT. This technique helps in reducing the residual risk of transfusion-transmitted HBV infection in HBsAg-negative blood donations in regions with low/high prevalence of HBV^10,31,33^ The approximate rate of HBV-NAT yield in the present work is 1:2000. The reported NAT yield for HBV was highly variable between different countries. It was low in USA and Europe (1:600,000–1:350,000), Kuwait (1:24,275), and South Africa (1:52,303). On the other hands, it was high in Ghana (1:232), Iran (1:125), Mozambique (1:69), Mexico (1:865), and Egypt (1:2609)^32^.

Mabunda et al.^11^ investigated the prevalence of HBV by NAT in serological negative blood samples from donors in blood bank of south and central Mozambique and the prevalence was 12.5 per 1000 seronegative blood units. Atef & Atef^10^ screened 34,671 seronegative HBs antigen samples by NAT and 14 samples were reactive. The 14 samples had a low viral load, and they documented the importance of NAT screening to reduce the potential risk of HBV transfusion-transmission and the necessity to implant the NAT screening in all blood banks in Egypt.

Occult HBV infection (OBI) was defined as HBV-DNA positive/HBs antigen negative with or without HBc antibodies or HBs antibodies. There are two types of OBI, seropositive (positive anti-HBc with or without anti-HBs) and seronegative (negative anti-HBc and anti-HBs). Many published papers stated that the most detected marker in OBI is the anti-HBc and recommended it to be included in screening of HBs antigen units^34,36^.There are 32 NAT positive/CLIA negative units in the present work. Can we consider them OBI?, are there any significance for testing such units for HBc antibodies? in the era of NAT (i.e. exclude all positive HBV-DNA units and thereby increasing the safety of transfusions) and its implementation in many blood banks in Egypt, is it needed to include HBc antibody in the screening guidelines?. Authors think that if safety is the major concern and the NAT is available, such positive units will be discarded, and safe units only will be transfused.

As regards the HIV CLIA results, 22 samples (0.03%) were reactive from a total of 87,620 samples, 16 (0.02) samples were from Assiut, and 6 (0.05%) samples were from Mansoura. Few reports were published on the prevalence of HIV among voluntary blood donors in Egypt. Senosy^37^, work on Beni-Suef University Hospital Blood Bank reported a prevalence of 0.1% HIV positive samples by ELISA (14 from 26,442 samples). Ebeid et al.^14^ reported that only 4/1000 samples (0.4%) were positive for HIV by ELISA. The prevalence of HIV in Egypt was reported to be less than 0.01% and it is mainly concentrated among intravenous drug abuser and homosexual men^37^. However, the incidence is increasing rapidly with about 25–30% annually over the last 10 years^38,41^. Gadji et al.^8^ compared the results of HIV testing by CLIA versus immunochromatographic and EIA tests and reported the HIV positivity in 328 donors by CLIA. The results of other techniques revealed positive results in negative cases in 115 donors and 15 discordant results. They recommended the importance of introducing the CLIA for screening of TTIs to enhance the safe blood supply in improve blood supply in low-income countries.

HIV-NAT reactive results in the present work were 19 samples (14 from Assiut and 5 samples from Mansoura) instead of 22 by CLIA. The TP samples were 19 (14 from Assiut and 5 from Mansoura), the FP (positive CLIA and negative NAT) samples were 3 (2 from Assiut and one sample from Mansoura) and no samples were reported as FN (negative CLIA and positive NAT). Ebeid et al.^14^ reported one (0.1%) HIV sample as TP (positive ELISA and NAT) and one (0.1%) sample as FN (negative ELISA and positive NAT). The HIV-NAT yield was zero in Pakistan^42^, 1:1250 in India^43^, 1.45 per million in China^44^, 0.43 per million in USA^32^ 1.8 per million in Italy^13^,0.43 per million in Germany^45^, and was high in South Africa (25.56 per million)^46^ and 2.6 per 1000 in seronegative units in Mozmpque^11^ Dubravac et al.^47^ compared the result of Architect i1000SR HIV Ag/Ab Combo in HIV with western blot and NAT assays and revealed that out of 2037 patients’ samples, 13 samples were TP, 3 samples FP and 2021 TN with no FN result. The low prevalence in Egypt can be explained by pre-donation donor questionnaire and examination which implicated in our two main blood centers and applied on all blood donor volunteers and the use of Architect i2000SR HIV Ag/Ab Combo (4 th generation) with higher sensitivity of early detection of HIV infection.

Although Egypt is an endemic area in HCV infection, however, the screening for HCV in Egyptian blood banks is mainly based on serological testing. Only limited centers in Egypt have recently implemented NAT for the screening of blood donations^48^ HCV reactivity by CLIA was detected in 1477/1.69% of the total 87,620 samples tested (1298/1.73% from Assiut and 179/1.44% from Mansoura). Testing the same samples by NAT revealed HCV reactivity in 1328/1.52% samples (1168 from Assiut and 160 from Mansoura). Comparing the results of CLIA with the results of NAT revealed the presence of TP result in 1305 units (positive CLIA and NAT), FP results in 172 units (positive CLIA and negative NAT), and FN results in 23 units (negative CLIA and positive NAT). This means that the use of NAT saved 172 from destruction and saved to be transfused (FP) and removed 23 units that were miss passed from CLIA (FN). Comparison of results of CLIA and NAT revealed 98.27% sensitivity, 99.8% specificity, and very good (0.929) Kappa coefficient value.

Egypt was the considered the country with the highest HCV prevalence in the world (15%) with 5–28% HCV seroprevalence among blood donors. However, infection rates have declined in the 21 st century^49^ Ebeid et al.^14^ reported the rate of 2.2% and 0.9% of HCV reactivity by ELISA and NAT respectively. El ekiaby et al.^50^ reported the prevalence of 2.6–4.5% and 1:3100 to 1:9500, of total HCV infections and NAT-yield respectively. Ibrahim et al.^48^ works on 456 samples from 2016 to 2018, reported a higher prevalence of HCV in blood donors (13.0%) by CLIA and 9.0% by ELISA. The countrywide campaign to eliminate HCV was started in 2014 and reinforced in 2018 and helped in reducing the prevalence of HCV from 10.0% to 0.38 over a decade. This study believes in the importance of improving the screening methods to effectively exclude as many high-risk blood donors as possible. Using NAT testing, we reported a high frequency of HBV and HCV in blood donors approved for blood donation by the routine screening process.

HCV-NAT yield (false seronegative and NAT reactive) rate was high (1:3000) in the present work. This yield is much higher than other Mediterranean countries such as Spain 2.15 per million, Greece 5.97 per million, Italy 2.5 per million, and Slovenia 4.27 per million^13,52,53^, and it was 2.6 per 1000 in seronegative units in Mozmpque^11^ However, in Egypt, the NAT yield donations was varied from 1:3100 to 1:9500^51^.

The scanning of HBV, HCV, HIV by both CLIA and NAT techniques revealed a perfect agreement regarding HBV with (kappa = 0.907), HCV (kappa = 0.929) and HIV, (kappa = 0.900). In line with these data Jin-feng et al.^29^ documented that the consistency of both serological assay and NAT was very good for HBV with (kappa = 0.815), fair for HCV with (kappa = 0.484) and good for HIV with (kappa = 0.608). Kouyoumjian et al.^54^ found that the NAT testing is important for detecting the FN units and preventing the release of such units for use and to help in more safe blood transfusion.

Although about 80% of the population are living in low to middle income countries, they receive only 20% of the worldwide safe blood, especially in Africa^8^ NAT implementation in routine screening for safe blood transfusion, is it mandatory and is it cost benefit? It is difficult to answer such a question, and its value varies from country to another depending on several factors, either economic or political. Egypt guidelines for blood transfusion, the NAT is not mandatory, although, many blood banks already have the NAT. Authors think that there are two situations that should be considered. First, false positive results, with serological assay, will be associated with the loss of the cost of blood units which will be discarded, the cost of screening tests in addition to decreasing the pool of blood donors. Second, false negative results will be associated with transfer of TTIs with infected blood units with the possibility of community spreading such infection in addition to the necessity to treat such patients and the burden of treatment and disability of chronic patients on the economy of their countries. Therefore, authors are convinced by the mandatory need of NAT as confirmatory screening tests for safe blood transfusion with comparable cost benefit.

The present study recommends the use of NAT assay in narrowing viral window phase. When NAT is combined with CLIA assay, the accuracy of results will be increased and the number of false reactive donations that discarded will be decreased. Also, it will increase the safety of blood transfer by detecting seronegative donations that pass or undetected by routine serological assay. Therefore, combining NAT and CLIA might be considered cost-effective and more liable to achieve the target of WHO for safe blood transfusion and zero risk transmission of TTIs.

## Conclusion

NAT implementation with CLIA increased blood transfusion safety with the advantage of direct sequence specific detection of virus genome. Therefore, it is a critical role to adopt NAT technology in all blood transfusion centers in Egypt.

## Data Availability

Data availabilityThe data underlying this article will be shared on reasonable request to the corresponding author.

## Abbreviations

NAT: Nucleic acid technique
TTIs: transfusion transmitted infections
CLIA: chemiluminescence
HBV: Hepatitis B virus
HCV: Hepatitis C virus
HIV: Human immune deficiency virus
TP: Treponema Pallidum
AIDS: acquired immunodeficiency syndrome
WHO: World Health Organization
TP: True positive
TN: True negative
FP: False positive
FN: False negative
PPV: Positive predictive value
NPV: Negative predictive value
